# Mega-Event Appraisals, Support, and Engagement Intentions Beyond Host Regions: Pre-Event Evidence from Two Non-Host Cities

**DOI:** 10.3390/bs16101727

**Published:** 2026-09-23

**Authors:** Zhuorui Jiang, Siyan Liu, Qian Gu

**Affiliations:** 1 School of Business, Xianda College of Economics & Humanities, Shanghai International Studies University, No. 390 Dongtiyuhui Road, Hongkou District, Shanghai 200080, China; jiangzhuoruihh@163.com; 2 School of Psychology, Shanghai University of Sport, No. 650 Qingyuan Ring Road, Yangpu District, Shanghai 200438, China; lsy13940650360@outlook.com

**Keywords:** sport mega-events, behavioral intentions, event involvement, social identity, social exchange, resident support, non-host residents

## Abstract

Using survey data collected in 2020 before Beijing 2022, this study examined associations among event involvement, perceived benefits and costs, hosting support, and engagement intentions among 2021 residents of Shanghai and Shenyang, two cities that did not host Beijing 2022 competitions. Ordered-categorical, weighted least squares mean- and variance-adjusted structural equation modeling and multi-group comparisons were applied. Event involvement was positively associated with local-instrumental benefits (β = 0.624) and symbolic-social benefits (β = 0.546) and negatively associated with perceived costs (β = −0.284). Symbolic-social benefits were associated with greater hosting support (β = 0.359), perceived costs with lower support (β = −0.120), and support with engagement intentions (β = 0.416). Event involvement (β = 0.406) and winter-sport involvement (β = 0.214) also had direct associations with engagement intentions. The local-instrumental-benefit association was small (β = 0.060) and sample-sensitive. False-discovery-rate-adjusted indirect associations involving support were significant for symbolic-social benefits (β = 0.149) and costs (β = −0.050). City differences were selective. The findings distinguish overall hosting support from intended engagement, while the cross-sectional design and single-item support measure limit causal interpretation.

## 1. Introduction

Although recent mega-event research increasingly focuses on longer-term legacy, pre-event appraisals remain important for understanding how residents assess expected benefits, costs, and opportunities before event delivery. This study focuses on residents outside competition sites and examines relationships among event involvement, perceived benefits and costs, hosting support, and intended engagement. Recent research distinguishes material burdens from social, psychological, and image-related returns in residents’ evaluations of major sport events (Liang et al., 2026; Matsuoka et al., 2025; Reimers et al., 2025; Solberg & Denstadli, 2026).

Beijing 2022 provides a useful setting for examining these relationships. Although competition was concentrated in designated host zones, the event was linked to wider ambitions for winter-sport development and national image across China (S. Chen et al., 2021). Resident-impact research has concentrated mainly on host communities, where construction, congestion, public spending, and local celebration make the exchange especially visible (Ap, 1992; Gursoy & Kendall, 2006; S. S. Kim & Petrick, 2005). Non-host responses, however, have also been documented: Deccio and Baloglu (2002) examined spillover responses outside the Salt Lake City host community, and later Olympic studies showed that perceived value, expected impacts, and legacy can extend beyond the principal host city (J. T. Bakhsh et al., 2024; Liu et al., 2017; Ritchie et al., 2020). Research on Beijing 2022 identified regional variation in expected impacts, perceived value, and behavioral intentions (Xu et al., 2022), while a later two-wave study documented changes in psychic income, support, and word-of-mouth intentions among host residents (Hou et al., 2026). Residents outside competition sites can therefore form appraisals and engagement intentions even without direct contact with event venues.

Favorable impact perceptions, event support, engagement intentions, and behavior should not be treated as interchangeable. Support is an evaluative judgment about whether hosting is legitimate or desirable, whereas engagement intentions concern prospective actions such as watching, attending, recommending, volunteering, or purchasing. The theory of planned behavior (TPB) positions intention close to behavior while specifying attitudes, subjective norms, perceived behavioral control, and correspondence with a particular action (Ajzen, 1991, 2020). Here, TPB is used to distinguish event-level evaluation from prospective engagement rather than as a complete behavioral model.

To explain how appraisal may connect with these responses, this study combines social exchange and social identity perspectives. Local-instrumental benefits concern tourism, regional development, environmental improvement, and infrastructure that residents may associate with practical opportunities. Symbolic-social benefits concern collective psychological gains, international exchange, national image, and sport development. Social exchange theory explains how anticipated returns and perceived costs enter event evaluation; social identity theory helps explain why collective image and pride may matter even when direct material returns are limited. This distinction provides an organizing framework for the heterogeneous benefit domains examined here (J. Bakhsh et al., 2018; Kaplanidou et al., 2013; Parra-Camacho et al., 2023).

Against this background, the study examines residents of Shanghai and Shenyang, neither of which hosted Beijing 2022 competitions. Existing studies document non-host spillovers, involvement–appraisal links, and associations between perceived impacts and support. The present study extends this literature by testing whether local-instrumental and symbolic-social appraisals relate differently to hosting support and broader engagement intentions, and by examining whether these associations vary across two non-host city samples using ordered-categorical models and formal between-city comparisons.

## 2. Theoretical Background and Hypotheses

### 2.1. Appraisal and Social Exchange Beyond the Host City

Social exchange theory proposes that residents evaluate an event by comparing anticipated returns with perceived burdens and favor the exchange when expected value is positive (Ap, 1992; Gursoy & Kendall, 2006). For host communities, this evaluation may include economic opportunity, social pride, congestion, public expenditure, safety, and environmental risk (H. J. Kim et al., 2006; Ohmann et al., 2006; Waitt, 2003). A recent meta-analysis found that perceived benefits were consistently associated with resident support, whereas pooled cost effects were more variable and depended on impact domain, event type, region, and event stage (Liang et al., 2026). For non-host residents, the exchange is more indirectly experienced: national narratives, sport-development initiatives, destination images, and imagined local opportunities may be salient even without daily contact with venue construction or neighborhood disruption.

Social identity theory complements the exchange account by explaining why favorable group image, pride, and shared meaning can have psychological value even when direct material returns are limited (Tajfel & Turner, 1979). Mega-event research has linked event identification and community pride with residents’ social responses and quality of life (Inoue et al., 2023; Magno & Dossena, 2020). The two perspectives therefore address different parts of the argument: social exchange concerns how perceived benefits and costs enter evaluation, whereas social identity helps interpret why symbolic benefits may be valued. In the present study, social identity provides an interpretive account of symbolic-social benefits rather than an independently tested mechanism.

### 2.2. Event Involvement and Impact Appraisal

Event involvement reflects the personal relevance of, interest in, and attention devoted to an event. Greater involvement is likely to correspond to greater exposure to information about tourism, infrastructure, national image, international exchange, and sport participation. Sport-event research has associated involvement with social-impact appraisals and event support, while recent winter-event evidence shows that the relevance of particular impacts can vary with event-related behavior (Helsen et al., 2022; Matsuoka et al., 2025; Taks & Rocha, 2022). On this basis, event involvement is expected to correspond to higher benefit appraisals and lower cost appraisals.

**H1.**  *Event involvement is positively associated with local-instrumental and symbolic-social benefit appraisals and negatively associated with perceived costs.*

### 2.3. Appraisal, Support, and Engagement Intentions

Symbolic-social benefits concern collective meaning: whether an event strengthens national image, promotes international exchange, advances sport, and generates psychological or social gains. Recent studies likewise show that civic pride, image enhancement, and psychic income can contribute to favorable event evaluation even when financial concerns remain visible (Matsuoka et al., 2025; Reimers et al., 2025; Solberg & Denstadli, 2026). From a social identity perspective, these appraisals can affirm positively valued group attributes; from a social exchange perspective, they constitute nonmaterial returns (Inoue et al., 2023; Magno & Dossena, 2020; Tajfel & Turner, 1979). Both perspectives imply that symbolic-social benefits should be associated with the perceived legitimacy and desirability of hosting, whereas perceived costs should signal a less favorable exchange.

**H2.**  *Symbolic-social benefits are positively associated with hosting support, whereas perceived costs are negatively associated with it.*

A global hosting-support judgment and engagement intentions are expected to be related without being equivalent. Endorsing an event may correspond to greater readiness to watch, attend, recommend, volunteer, or purchase, but each action also depends on opportunity and motivation. Consistent with the TPB distinction between evaluation and intention (Ajzen, 1991, 2020), hosting support is treated as an evaluative judgment associated with engagement intentions rather than as the behavioral outcome itself.

Local-instrumental benefits may also be associated with engagement through perceived utility. Anticipated tourism, regional development, environmental improvement, and infrastructure can make event-related actions appear worthwhile even after general support is held constant. Research on sport-event tourism similarly connects perceived local impacts and community participation with behavioral support for event development (Boonsiritomachai & Phonthanukitithaworn, 2019).

### 2.4. Event and Sport Involvement

A global hosting-support judgment is unlikely to account fully for engagement. Following event news, considering the event personally important, and identifying with winter sport may be associated with intended action independently of that judgment. Event involvement captures proximity to a specific event, whereas winter-sport involvement captures a broader sport-based predisposition. This distinction is relevant because exposure to elite sport does not automatically generate participation without personal relevance and accessible opportunities (Weed et al., 2015).

**H3.**  *Hosting support, local-instrumental benefits, and event- and sport-specific involvement are positively associated with event-related engagement intentions.*

### 2.5. Indirect Associations Involving Hosting Support

The combined framework also implies indirect associations involving hosting support. Symbolic-social benefits may be related to engagement partly through their association with hosting support, whereas perceived costs may show the opposite pattern. Product-of-coefficients tests are used to evaluate these model-implied associations.

**H4.**  *Symbolic-social benefits have a positive model-implied indirect association with engagement intentions involving hosting support, whereas perceived costs have a negative indirect association involving support.*

### 2.6. Exploratory Regional Comparison

Regional context may condition selected associations. Shanghai and Shenyang provide two contrasting non-host settings in which winter-sport familiarity, event exposure, and local opportunity structures may differ. Because existing theory does not justify directional predictions for every structural path, the between-city analysis is treated as exploratory. RQ1. Do the structural pathways differ between residents of Shanghai and Shenyang? Figure 1 presents the conceptual model.

## 3. Materials and Methods

### 3.1. Study Design, Recruitment, and Participants

A cross-sectional online survey was conducted among residents of Shanghai and Shenyang, two cities that did not host Beijing 2022 competitions. Fieldwork ran from October to December 2020 through the Wenjuanxing sample-service channel. The nonprobability online panel recruited residents of the two cities who could complete the Chinese-language questionnaire. City assignment used location metadata attached to the platform record; self-reported district and duration of residence were also collected. The platform provided 2044 completed questionnaires (Shanghai, *n* = 1020; Shenyang, *n* = 1024). Counts of invitations, landing-page views, partial starts, and withdrawals were not retained, so a conventional response or completion rate cannot be calculated.

Participation was voluntary. Electronic informed consent was obtained from adult participants before questionnaire access. For respondents younger than 18 years, permission from a parent or legal guardian and informed assent from the respondent were obtained before participation. The analytic questionnaire did not request names or contact details. Internet-protocol metadata used for location verification was retained only in the restricted raw export and removed from the de-identified analytic file.

Data screening identified 23 respondents who selected the same response option across all 83 Likert-type items. These cases were excluded because the pattern provided no within-person differentiation across substantively diverse measures. No retained respondent had an exactly duplicated 83-item response vector, and no case had missing values on the focal items. Internet-protocol addresses were not used as an exclusion criterion because shared household, institutional, or mobile networks can legitimately generate repeated addresses. The final analytic sample comprised 2021 respondents: 1015 from Shanghai and 1006 from Shenyang. Women represented 58.9% of the sample, 47.2% were aged 18–30 years, 65.3% held a bachelor’s degree, and 44.2% identified as winter-sport enthusiasts. Sample characteristics are summarized in Table 1.

Data from the same 2020 survey project were previously analyzed by Jiang et al. (2021) in relation to social cohesion. The present study examines hosting support and engagement intentions; details of sample and measure overlap are provided in Supplementary Note A.

### 3.2. Instrument Development and Response Format

Questionnaire development was informed by established research on residents’ event-impact appraisals and support (Ap, 1992; Gursoy & Kendall, 2006; S. S. Kim & Petrick, 2005; Liu et al., 2017). After an initial literature-based item pool was drafted, two structured resident focus groups were conducted for instrument refinement: 10 adults in Shenyang on 17 February 2019 and 10 adults in Shanghai on 5 March 2019. Participants were publicly recruited through a survey-research company, with selection intended to provide variation in sex, age, occupation, education, and residence duration. Discussions covered event awareness and involvement, winter-sport experience, anticipated positive and negative effects, and overall hosting support. Their input added or clarified locally salient content, including international exchange, facility use, public expenditure, and safety and health concerns.

A 110-person Shanghai pilot was subsequently used for item discrimination, exploratory factor analysis, and reliability checks. One positive-impact item and one safety item were removed; the retained instrument comprised 31 benefit and 15 cost items. Supplementary Table S1 lists each focal item, its construct assignment, and its documented development source. Unless otherwise stated, items were rated from 1 (strongly disagree) to 5 (strongly agree).

### 3.3. Measures

#### 3.3.1. Event Involvement

Event involvement was measured with six items assessing the personal importance, interest, information seeking, and value relevance of following Beijing 2022 (e.g., “I closely follow information about the Beijing Olympic Winter Games”). The six-item scale showed good internal consistency in the cleaned sample (Cronbach’s α = 0.875).

#### 3.3.2. Winter-Sport Involvement

Winter-sport involvement was assessed with two items: identification as a winter-sport enthusiast and regular participation in winter sports. The items were strongly related (α = 0.852) and were averaged as an observed variable. Keeping them separate from event involvement was supported by the two-factor measurement model. Because two indicators cover only identity and participation frequency, this measure represents a narrow sport-involvement index rather than the full cognitive, affective, and behavioral breadth of involvement.

#### 3.3.3. Perceived Benefits

Thirty-one items assessed seven first-order positive-impact dimensions: tourism (4 items), economic and environmental development (5), social capital and psychological gains (7), international cooperation and exchange (4), infrastructure (3), national image (4), and sport development (4). In the compact model, the three local dimension scores served as representative continuous indicators of local-instrumental benefits, and the four symbolic dimension scores served as indicators of symbolic-social benefits. The local-versus-symbolic grouping was a theory-informed analytic organization evaluated with the present data. Separate ordinal item-level confirmatory factor analyses (CFAs) evaluated the underlying structure. The two-domain higher-order solution had borderline incremental fit (CFI = 0.900; TLI = 0.890; RMSEA = 0.070; SRMR = 0.060), so the domains are treated as parsimonious summaries. First-order reliabilities ranged from α = 0.741 to 0.855.

#### 3.3.4. Perceived Costs

Fifteen items assessed living and resource costs (6 items), safety risks (3), pollution risks (3), and disease or health risks (3). The four dimension scores served as representative continuous indicators of aggregate perceived costs in the compact model; first-order reliabilities ranged from α = 0.780 to 0.829. The item-level ordinal higher-order model showed weak absolute fit (CFI = 0.937; TLI = 0.923; RMSEA = 0.110; SRMR = 0.054). Sensitivity analyses therefore also examined the separate cost dimensions and an aggregate factor omitting health risk.

#### 3.3.5. Hosting Support

Hosting support was assessed with the direct item, ‘I support hosting the Beijing Olympic Winter Games,’ and analyzed as a single observed ordered variable. Two additional questionnaire items assessed whether benefits exceeded costs and whether hosting was a waste of money. After reverse-scoring the latter, the three-item composite had low internal consistency (α = 0.540). The direct item was therefore retained as the clearest measure of overall hosting support, and sensitivity analyses examined both the broader composite and a three-item ordered latent factor.

#### 3.3.6. Event-Related Engagement Intentions

Engagement intentions were measured with five items concerning watching television coverage, attending in person, recommending attendance, volunteering, and purchasing event-related souvenirs (α = 0.831). The items were modeled as a one-factor construct, but they differ in opportunity, cost, target, and behavioral specificity. Consistent with the TPB compatibility principle (Ajzen, 2020), the factor is interpreted as a general propensity toward event-related engagement rather than a precise intention to perform one behavior or as evidence of actual winter-sport participation.

### 3.4. Statistical Analysis

Analyses were conducted in R 4.4.1 (R Core Team, 2024) using lavaan 0.6-19 (Rosseel, 2012). Initial ordinal measurement comparisons evaluated alternative structures for involvement, benefits, costs, and engagement. In the compact model, the six event-involvement items, global hosting-support item, and five engagement items were treated as ordered five-category variables and analyzed using weighted least squares mean- and variance-adjusted estimation (WLSMV; theta parameterization). Winter-sport involvement was the mean of its two items. Seven benefit and four cost dimension scores were treated as continuous domain-representative indicators of local-instrumental, symbolic-social, and perceived-cost factors. Full item wording, alternative specifications, and executable analysis syntax are provided in Supplementary Files S1 and S2.

Multi-group measurement invariance across Shanghai and Shenyang was examined sequentially for the five multi-indicator factors at the configural, metric, scalar/threshold-intercept, and strict levels. Event-involvement and engagement items were ordered; benefit and cost dimension-score indicators were continuous. Models used theta parameterization, mean structures, and lavaan’s default marker-indicator identification (the first loading of each factor fixed to 1 in the configural model). Equality constraints were added to loadings; then to ordered-item thresholds and continuous-indicator intercepts; and finally to residual variances. In the city-specific structural model, the thresholds of the observed ordered support item were also constrained equally. Factor means and variances followed lavaan’s identification rules for each invariance stage. Exact syntax and parameter-handling details are reproduced in Supplementary Methods S1.

The structural model regressed local-instrumental benefits, symbolic-social benefits, and perceived costs on event involvement and winter-sport involvement. Hosting support was regressed on symbolic-social benefits, perceived costs, event involvement, and winter-sport involvement. Engagement intentions were regressed on hosting support, local-instrumental benefits, event involvement, and winter-sport involvement. Event involvement was allowed to covary with winter-sport involvement, and the three perceived-impact constructs were allowed to covary. Model fit was evaluated using scaled chi-square, CFI, Tucker–Lewis index (TLI), RMSEA, and SRMR. Conventional guidelines were used as approximate indicators rather than mechanical cutoffs (Hu & Bentler, 1999).

Indirect associations were calculated from labeled WLSMV paths using delta-method standard errors and 95% confidence intervals (CIs); Benjamini–Hochberg correction was applied across the five indirect tests. For measurement invariance, a decrease in CFI greater than 0.010 or an increase in RMSEA greater than 0.015 was treated as evidence against invariance (F. F. Chen, 2007). City-specific coefficients were estimated after the latent measurement model satisfied these incremental-fit criteria. Regional differences were tested by constraining one structural path at a time and comparing each nested model with the freely estimated multi-group model using lavaan’s WLSMV scaled Satorra (2000) difference test; false-discovery-rate correction was applied across all 14 structural comparisons. Convergence and admissibility were checked for every reported model. Sensitivity analyses examined alternative support specifications, separate first-order benefit and cost dimensions, an aggregate cost factor without health risk, suppression diagnostics, re-estimation with all 2044 completed questionnaires, and an age-restricted sample. An unrotated first-component diagnostic was also calculated across the 60 focal items. No prospective power calculation was conducted; all completed questionnaires available within the planned field period were retained after the stated quality screen.

## 4. Results

### 4.1. Preliminary Analyses and Descriptive Statistics

The final sample included 2021 respondents. Table 2 presents descriptive statistics and zero-order correlations for the focal scale and domain scores. Mean scores were relatively high for symbolic-social benefits and hosting support, while perceived costs were below the scale midpoint. Event involvement showed its largest zero-order correlation with engagement intentions, followed by local-instrumental benefits and winter-sport involvement.

### 4.2. Measurement Structure and Invariance

Ordinal measurement comparisons favored treating event involvement and winter-sport involvement as separate constructs. The eight-item one-factor model fit poorly (CFI = 0.908; TLI = 0.871; RMSEA = 0.218; SRMR = 0.099), whereas the correlated two-factor model fit substantially better (CFI = 0.986, TLI = 0.979, RMSEA = 0.087, SRMR = 0.035). Engagement intentions were adequately represented by one factor (CFI = 0.994; TLI = 0.988; RMSEA = 0.070; SRMR = 0.023). The seven-correlated-benefit-factor model (CFI = 0.904; RMSEA = 0.070; SRMR = 0.057) and two-higher-order-domain model (CFI = 0.900; TLI = 0.890; RMSEA = 0.070; SRMR = 0.060) showed borderline incremental fit. Item-level four-factor and higher-order cost models had poor absolute fit (higher-order CFI = 0.937; TLI = 0.923; RMSEA = 0.110; SRMR = 0.054). Accordingly, the higher-order structures are used as compact domain summaries and are interpreted alongside the first-order sensitivity analyses.

The compact measurement model satisfied the incremental-fit criteria at the configural, metric, scalar/threshold-intercept, and strict levels across Shanghai and Shenyang. CFI values were 0.931, 0.939, 0.932, and 0.935, respectively; RMSEA values were 0.062, 0.057, 0.058, and 0.055; and SRMR values ranged from 0.040 to 0.043. No step decreased CFI by more than 0.007 or increased RMSEA beyond the stated 0.015 criterion. Robust scaled chi-square difference tests were statistically significant for configural versus metric, Δχ^2^(17) = 72.50, *p* < 0.001; metric versus scalar, Δχ^2^(39) = 123.30, *p* < 0.001; and scalar versus strict, Δχ^2^(22) = 48.49, *p* < 0.001. Given the large sample, comparability was judged from the incremental-fit criteria together with the full pattern of fit indices rather than chi-square alone. All models converged and passed lavaan’s post-estimation admissibility check. Complete results and syntax are provided in Supplementary Table S2, Supplementary Methods S1, and Supplementary File S2. Table 3 summarizes the measurement-invariance results.

### 4.3. Structural Model Fit and Explained Variance

The compact ordered-categorical structural model showed adequate approximate fit for the domain-score specification; scaled χ2(236) = 2030.92; CFI = 0.925; TLI = 0.912; RMSEA = 0.061; and SRMR = 0.038. It explained 37.8% of local-instrumental benefits, 25.2% of symbolic-social benefits, 5.9% of perceived costs, 25.2% of the latent response underlying the observed support item, and 68.4% of engagement intentions. Because the item-level higher-order benefit and cost structures were weaker, the structural findings are interpreted together with the measurement and sensitivity analyses.

### 4.4. Overall Structural Pathways

Table 4 reports the overall structural coefficients. Event involvement was positively associated with local-instrumental benefits (β = 0.624, *p* < 0.001) and symbolic-social benefits (β = 0.546, *p* < 0.001) and negatively associated with perceived costs (β = −0.284, *p* < 0.001), supporting H1. Symbolic-social benefits were positively associated with hosting support (β = 0.359, *p* < 0.001), whereas aggregate perceived costs were negatively associated with support (β = −0.120, *p* < 0.001), supporting H2 at the aggregate level. Event involvement also retained a positive adjusted association with support (β = 0.188, *p* < 0.001).

Hosting support was positively associated with engagement intentions (β = 0.416, *p* < 0.001). Event involvement (β = 0.406, *p* < 0.001) and winter-sport involvement (β = 0.214, *p* < 0.001) also retained direct associations with engagement. These results support three components of H3. Local-instrumental benefits had a smaller direct association in the primary sample (β = 0.060, *p* = 0.019); this estimate became nonsignificant in the age-restricted sensitivity model. Figure 2 displays selected standardized estimates from the primary model.

Sensitivity analyses generally preserved the central directions. Alternative support specifications produced similar focal coefficients, although the three-item latent model fit poorly. The aggregate cost–support association remained negative when health risk was omitted; when the cost dimensions were entered simultaneously, health risk had the clearest unique association with support. Conditional winter-sport coefficients showed sign reversals relative to their zero-order correlations and are therefore not interpreted substantively. Full results are reported in Supplementary Table S3.

First-order score models confirmed that event involvement was positively associated with all seven benefit scores and negatively associated with all four cost scores. When domains were entered simultaneously, social-capital/psychological gains and national image retained unique associations with support, while economic/environmental development and infrastructure retained unique associations with engagement. These patterns support cautious use of the compact domains while indicating heterogeneity among their components (Supplementary Table S5).

Sample-definition analyses preserved the principal appraisal, support, and involvement pathways in both the full completed sample (*N* = 2044) and the adult-only sample (*n* = 1935). The exception was the small local-instrumental-benefit-to-engagement association, which was nonsignificant in the adult-only sample (β = 0.041, *p* = 0.115; Supplementary Table S4). The first-component diagnostic accounted for 24.41% of variance but does not rule out common-method bias.

### 4.5. Indirect Associations

Table 5 reports the five tested model-implied indirect associations. Hosting support formed part of a positive indirect association between symbolic-social benefits and engagement intentions (standardized estimate = 0.149; b = 0.301, 95% CI [0.245, 0.356], pBH < 0.001) and a negative indirect association between costs and engagement (standardized estimate = −0.050; b = −0.089, 95% CI [−0.123, −0.054], pBH < 0.001), consistent with H4. Event involvement additionally had indirect associations involving symbolic-social benefits and support (β = 0.081), costs and support (β = 0.014), and local-instrumental benefits (β = 0.037). All five tests remained significant after Benjamini–Hochberg correction; the adjusted *p* value for the smallest estimate was 0.019.

### 4.6. Regional Differences in Structural Pathways

The multi-group WLSMV model retaining strict measurement constraints showed adequate approximate fit; scaled χ2(556) = 2296.74; CFI = 0.926; TLI = 0.927; RMSEA = 0.056; and SRMR = 0.044. Formal single-path equality constraints with false-discovery-rate correction indicated selective between-city heterogeneity (Table 6).

Event involvement was more strongly associated with local-instrumental benefits in Shanghai (β = 0.789) than in Shenyang (β = 0.501), pFDR < 0.001; the same pattern appeared for symbolic-social benefits (β = 0.666 vs. 0.481), pFDR = 0.042. Its negative association with costs was also stronger in Shanghai (β = −0.432 vs. −0.204), pFDR < 0.001. Costs were negatively associated with support in Shanghai (β = −0.190) but not in Shenyang (β = −0.025), and the between-city difference was reliable, pFDR < 0.001.

Support was more strongly associated with engagement in Shenyang (β = 0.505) than in Shanghai (β = 0.329), pFDR < 0.001, and winter-sport involvement also had a stronger engagement association in Shenyang (β = 0.279 vs. 0.139), pFDR = 0.023. Event involvement showed the reverse pattern (β = 0.508 vs. 0.335), pFDR = 0.024. The local-benefit-to-engagement association did not differ reliably, pFDR = 0.097, and the symbolic-benefit-to-support association was comparable, pFDR = 0.444. The remaining city differences involved conditional winter-sport paths with sign reversals; these are reported in Table 6. Overall, RQ1 indicates selective rather than uniform between-city heterogeneity.

### 4.7. Hypothesis Evaluation

Table 7 summarizes the conclusions for the four hypotheses and the exploratory research question.

## 5. Discussion

This study examined how involvement, impact appraisals, a global hosting-support judgment, and engagement intentions were related among residents of two cities outside the Beijing 2022 competition zones. The findings indicate that non-host responses are not reducible to a single favorable evaluation. Event involvement corresponded with benefit and cost appraisals; symbolic-social appraisals and aggregate costs were most clearly associated with the observed hosting-support item; and both support and involvement were associated with intended engagement. The city analysis identified selective differences in association strength, while the city label itself remains a contextual marker rather than an explanation for those differences.

### 5.1. Event Involvement and Impact Appraisals

Event involvement was associated with all three appraisal domains, consistent with research linking event or sport involvement to impact perceptions and support (Helsen et al., 2022; C. Kim & Kaplanidou, 2019). In non-host settings, such appraisals may arise less from direct contact with venue construction or visitor flows than from event information, sport interest, collective narratives, and anticipated opportunities. The findings therefore suggest that event relevance can extend beyond competition sites.

Because involvement and appraisal were measured concurrently, their direction of association remains uncertain: greater attention may shape event appraisals, while favorable or unfavorable appraisals may also influence subsequent attention.

### 5.2. Symbolic-Social and Local-Instrumental Benefits

Symbolic-social benefits were more consistently associated with hosting support than local-instrumental benefits were with engagement. This pattern accords with evidence that image enhancement, civic pride, collective meaning, and psychic income can matter alongside material returns (Cai, 2022; Gibson et al., 2014; Hou et al., 2026; Matsuoka et al., 2025; Reimers et al., 2025; Solberg & Denstadli, 2026). Social exchange theory treats these experiences as nonmaterial returns, while social identity offers an interpretation of why collective image and pride may matter even when direct local gains are limited. Because national, city, and event identification were not measured, the identity account remains interpretive rather than mechanistic.

The broader benefit categories were used as parsimonious summaries of theoretically related first-order dimensions rather than on the assumption that all constituent dimensions operate identically. Local-instrumental benefits summarize primarily place-based and practical returns, whereas symbolic-social benefits summarize more collective or representational returns. Consistent with this distinction, the first-order sensitivity analyses showed heterogeneity within the broader domains: social-capital/psychological gains and national image retained unique associations with hosting support, whereas economic/environmental development and infrastructure retained unique associations with engagement intentions. The broader categories should therefore be interpreted as organizing summaries rather than as homogeneous sets of effects.

The indirect association linking symbolic-social appraisal, hosting support, and engagement is consistent with this pattern. Recent two-wave Beijing 2022 research linked changes in psychic income with changes in support and word-of-mouth intention among host residents (Hou et al., 2026), while host and non-host studies show that event value can extend beyond the immediate venue city (J. T. Bakhsh et al., 2024; Liu et al., 2017; Ritchie et al., 2020). The weak and sample-sensitive local-instrumental route further indicates that the two benefit domains are not interchangeable.

Perceived costs were negatively associated with hosting support, consistent with social-exchange models in which endorsement is lower when the exchange is perceived as burdensome (Gursoy & Kendall, 2006; Gursoy et al., 2017; Parra-Camacho et al., 2023). Meta-analytic evidence likewise suggests that cost–support associations vary by cost domain and study context (Liang et al., 2026). In the sensitivity analyses, health risk showed the clearest unique association when correlated cost domains were entered together, but the aggregate cost association persisted when health risk was omitted; in the first-order score model, no separate cost retained a unique association. Taken together, the cost results were more consistent at the aggregate than at the individual-domain level.

### 5.3. Hosting Support and Engagement Intentions

Hosting support was associated with engagement intentions, but event and winter-sport involvement retained independent associations with engagement. This distinction is plausible because an overall hosting judgment differs from actions such as watching, attending, volunteering, recommending, and purchasing, each of which requires different motivation and opportunity. Comparable research has connected perceived value, psychic income, and overall attitudes with behavioral intentions or support (Hou et al., 2026; Reimers et al., 2025; Xu et al., 2022). These findings support separating event-level support from a broader engagement propensity. Given the single-item support measure and cross-sectional design, the indirect association should not be interpreted as a temporal endorsement-to-action process.

Event involvement and winter-sport involvement may represent different motivational resources: the former captures attention to Beijing 2022, whereas the latter reflects an existing, albeit narrowly measured, connection to winter sport. Research on the PyeongChang Winter Games has likewise reported associations among sport involvement, impact appraisal, and support (C. Kim & Kaplanidou, 2019). Sport involvement or event interest may increase readiness to engage, but participation also depends on accessible opportunities (Potwarka et al., 2020; Weed et al., 2015). The engagement factor should therefore be interpreted as a general intention construct rather than a proxy for behavior.

### 5.4. Exploratory Regional Heterogeneity

The measurement model satisfied the incremental-fit criteria through the strict level despite significant robust chi-square difference tests, supporting cautious comparison of city-specific associations. Event-specific attention was more strongly related to several appraisal and engagement paths in Shanghai, whereas support and winter-sport involvement were more strongly related to engagement in Shenyang. These differences indicate heterogeneity in association strength; they do not identify city membership as a causal explanation.

Earlier Beijing 2022 studies reported regional variation in perceived impacts and behavioral intentions (Xu et al., 2022), and research with Shanghai residents linked expected social impacts with attitudes toward the Olympic bid (Liu et al., 2017). The present city comparisons extend this literature by testing structural path differences after establishing comparable measurement across the two samples. The pattern also accords with broader evidence that host configuration and geographical position can shape residents’ event valuation (J. Bakhsh et al., 2018; J. T. Bakhsh et al., 2024; Ritchie et al., 2020).

Possible explanations for the city pattern include differences in winter-sport familiarity, facilities, climate, media exposure, and local policy opportunities, but these features were not measured directly. Future multi-city research should replace the city proxy with direct measures of access, culture, communication exposure, and policy contact.

### 5.5. Theoretical Implications

Substantively, the results reinforce the distinction between hosting support and engagement intentions. A resident may favor hosting without expressing the same readiness to watch, attend, volunteer, recommend, or purchase. The results also suggest that benefit content matters: symbolic-social appraisals were more consistently associated with hosting support, whereas the direct local-instrumental association with engagement was small and sample-sensitive.

Methodologically, establishing measurement comparability before testing city-specific paths strengthens the comparative interpretation. The observed heterogeneity identifies where associations differ, but not why; contextual explanations require direct measures of facility access, sport familiarity, media exposure, and policy contact.

### 5.6. Practical Implications

The findings suggest a practical distinction between communication aimed at event approval and opportunities intended to facilitate engagement. Event-leverage research similarly argues that desired social outcomes require planned programs rather than exposure alone (Chalip, 2006; Potwarka & Wicker, 2021).

For residents with stronger event or winter-sport involvement, organizers could evaluate accessible opportunities such as community viewing, introductory sport sessions, club activities, or volunteering. In non-host settings, communication can also make expected local relevance and potential costs more transparent.

The city differences also suggest that engagement strategies should be evaluated against directly measured contextual conditions, including sport familiarity, facility access, media exposure, and local policy opportunities. Two-way communication and credible public engagement may be particularly relevant when residents assess benefits and burdens without direct venue contact (S. Kim & Manoli, 2022).

Future evaluation should use behavior-specific outcomes because watching, attending, volunteering, purchasing, recommending, and taking up a sport involve different costs and opportunities. Campaign experiments, registrations, ticketing, facility entry, and follow-up surveys could test whether particular strategies translate favorable evaluations into observable participation.

### 5.7. Limitations and Future Research

First, the cross-sectional design cannot establish temporal or causal direction. The 2020 survey captures expected rather than experienced event impacts, and fieldwork occurred during the COVID-19 pandemic, which may have affected perceptions of health risk and the perceived feasibility of attendance or volunteering (Thomson et al., 2025). Longitudinal work spanning pre-event, event, and post-event periods would provide stronger evidence on how these relationships change over time.

Second, the outcome measures have important boundaries. Engagement intentions combine behaviors that differ in target, opportunity, and cost, and hosting support was measured with a single observed item. Although alternative support specifications produced a similar general pattern, future work should use behavior-specific outcomes and purpose-designed multi-item support measures.

Third, the nonprobability online sample limits population generalization, particularly to older and less-educated residents, and the two-city design cannot represent non-host regions more broadly. Future comparative studies should include more locations and directly measure the contextual characteristics currently represented only by city.

Fourth, all focal variables were collected through the same questionnaire at one time point. The first-component diagnostic did not indicate a dominant common factor, but such diagnostics cannot rule out common-method bias (Fuller et al., 2016; Podsakoff et al., 2003). Multi-source or temporally separated designs would strengthen future tests.

Finally, the local-instrumental versus symbolic-social grouping was evaluated in the present dataset rather than preregistered. The item-level higher-order structures were not uniformly strong, and the first-order sensitivity analyses showed heterogeneity across constituent dimensions. The broader categories should therefore be viewed as parsimonious organizing summaries that require replication with purpose-designed measures and independent samples.

## 6. Conclusions

Using 2020 survey data from two non-host cities, this study found that event involvement was associated with how residents appraised expected benefits and costs and with their intentions to engage with Beijing 2022. Symbolic-social benefits and aggregate perceived costs were associated with hosting support, while hosting support, event involvement, and winter-sport involvement were associated with engagement intentions. The direct local-instrumental-benefit association with engagement was small and sample-sensitive, and between-city differences were selective. Taken together, the findings support distinguishing event-level hosting support from broader engagement intentions and suggest that different appraisal domains may relate to these responses in different ways. Because the evidence is cross-sectional and hosting support was measured with a single item, the identified pathways should be interpreted as associations rather than causal or staged processes.

## Figures and Tables

**Figure 1:**
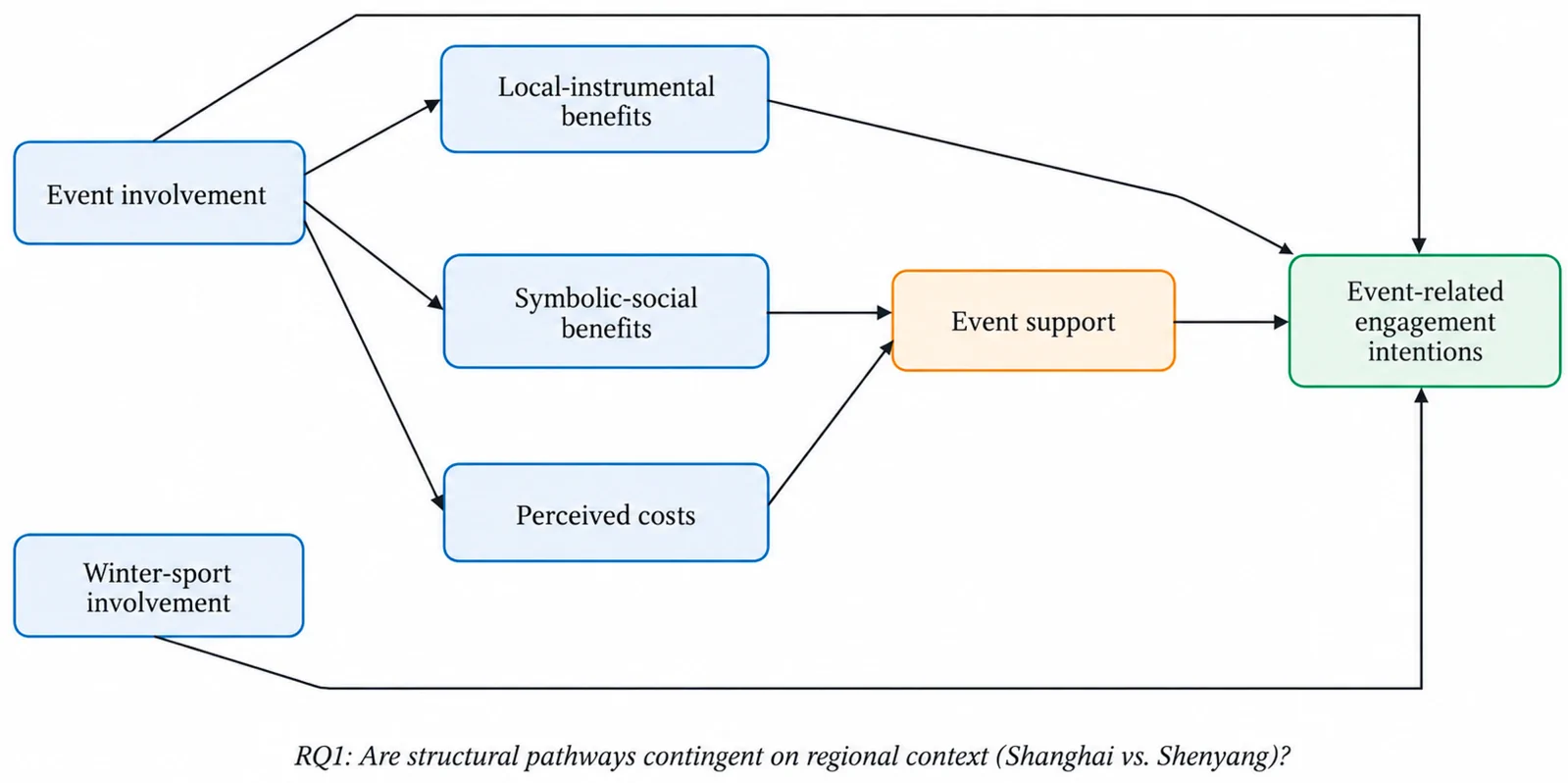
Conceptual model. Focal theory-guided pathways are shown; additional adjusted paths and covariances are described in the Methods. ‘Event support’ denotes the single global observed hosting-support item.

**Figure 2:**
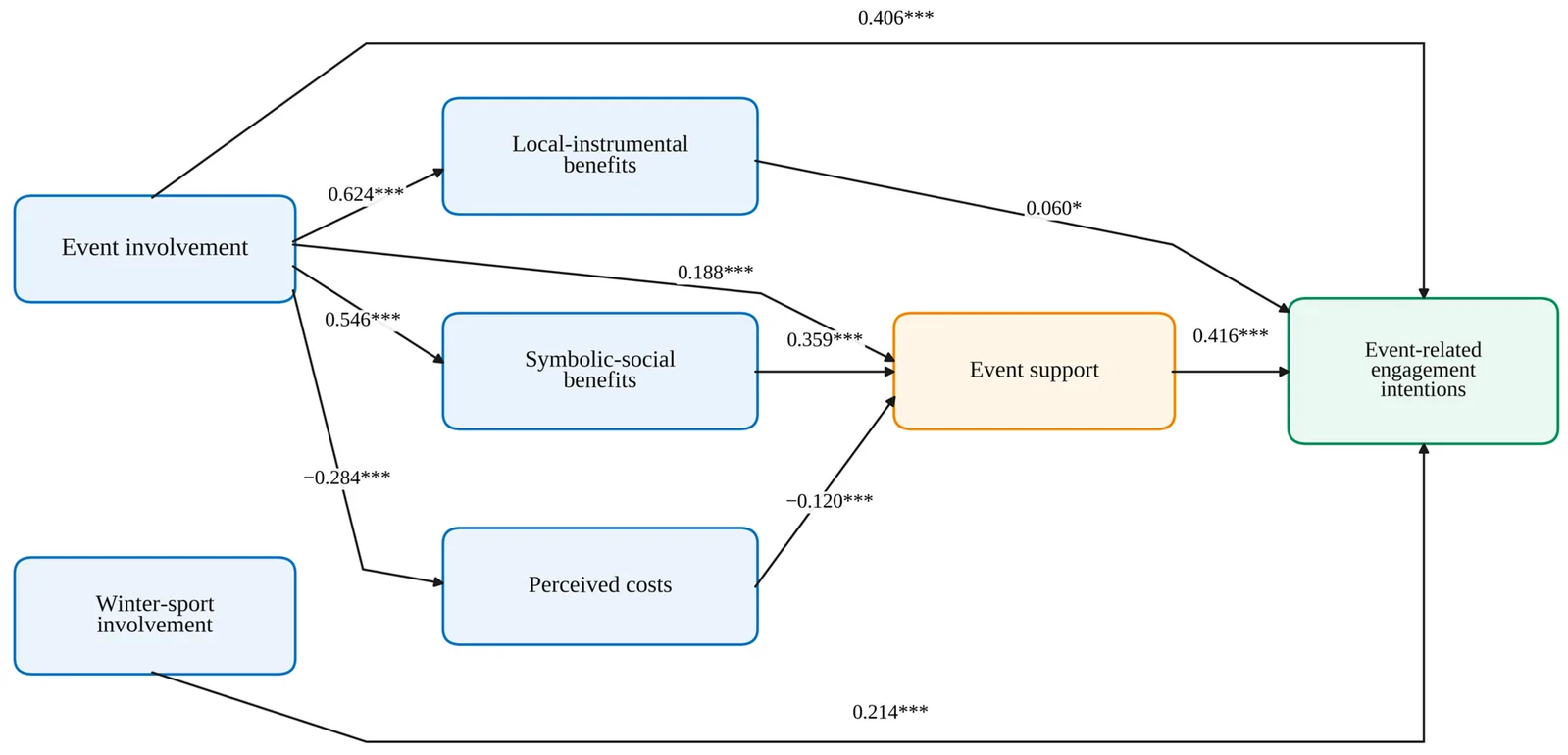
Selected standardized WLSMV estimates for the compact behavioral model. ‘Event support’ denotes the single global observed hosting-support item. Standardized coefficients are shown. * *p* < 0.05; *** *p* < 0.001. Small residual winter-sport paths are omitted for clarity.

**Table 1:** Sample characteristics (*N* = 2021).

Characteristic	Category	*n*	%
Gender	Male	830	41.1
	Female	1191	58.9
Age	<18 years	86	4.3
	18–30 years	953	47.2
	31–40 years	617	30.5
	41–50 years	254	12.6
	≥51 years	111	5.5
Education	Junior high or below	57	2.8
	High school/vocational	162	8.0
	College diploma	306	15.1
	Bachelor’s degree	1319	65.3
	Master’s degree or above	177	8.8
Monthly income	≤2999 RMB	584	28.9
	3000–3999 RMB	165	8.2
	4000–6999 RMB	384	19.0
	7000–9999 RMB	386	19.1
	10,000–14,999 RMB	336	16.6
	≥15,000 RMB	166	8.2
Winter-sport enthusiast	No	1128	55.8
	Yes	893	44.2

*Note.* Shanghai *n* = 1015; Shenyang *n* = 1006.

**Table 2:** Descriptive statistics and zero-order correlations.

Variable	M	SD	1	2	3	4	5	6	7
1. Event involvement	3.53	0.83	—						
2. Winter-sport involvement	2.84	1.18	0.538	—					
3. Local-instrumental benefits	3.74	0.72	0.546	0.321	—				
4. Symbolic-social benefits	4.28	0.53	0.438	0.201	0.546	—			
5. Perceived costs	2.73	0.80	−0.202	−0.072	−0.112	−0.183	—		
6. Global observed hosting support	4.25	0.91	0.304	0.112	0.238	0.410	−0.174	—	
7. Engagement intentions	3.65	0.82	0.613	0.478	0.430	0.410	−0.162	0.466	—

*Note*. *N* = 2021. M = mean; SD = standard deviation. Pearson correlations are based on observed scale or domain scores and are reported for descriptive purposes. An em dash on the diagonal indicates that a variable is not correlated with itself.

**Table 3:** Summary of multi-group measurement invariance.

Level	CFI	TLI	RMSEA	SRMR	ΔCFI	ΔRMSEA
Configural	0.931	0.920	0.062	0.040	—	—
Metric	0.939	0.932	0.057	0.043	+0.007	−0.005
Scalar/threshold-intercept	0.932	0.930	0.058	0.042	−0.007	+0.000
Strict	0.935	0.937	0.055	0.043	+0.004	−0.003

*Note*. WLSMV estimates are reported. Scalar equality jointly constrained thresholds for ordered indicators and intercepts for continuous dimension-score indicators. Δ indices were calculated from unrounded fit values. All changes satisfied the invariance criteria described in Materials and Methods. For the configural model, em dashes indicate that no preceding model is available for calculating incremental change.

**Table 4:** WLSMV pathways in the compact behavioral model.

Path	B	SE	β	*p*	95% CI for B
**Event involvement → local-instrumental benefits**	**0.281**	**0.017**	**0.624**	**<0.001**	**[0.248, 0.314]**
Winter-sport involvement → local-instrumental benefits	−0.009	0.013	−0.015	0.520	[−0.035, 0.018]
**Event involvement → symbolic-social benefits**	**0.194**	**0.012**	**0.546**	**<0.001**	**[0.171, 0.216]**
Winter-sport involvement → symbolic-social benefits	−0.038	0.013	−0.086	0.003	[−0.063, −0.013]
**Event involvement → perceived costs**	**−0.114**	**0.013**	**−0.284**	**<0.001**	**[−0.139, −0.089]**
Winter-sport involvement → perceived costs	0.046	0.013	0.091	<0.001	[0.021, 0.071]
Symbolic-social benefits → global observed hosting support	**0.790**	**0.056**	**0.359**	**<0.001**	**[0.680, 0.901]**
Perceived costs → global observed hosting support	**−0.233**	**0.046**	**−0.120**	**<0.001**	**[−0.323, −0.142]**
Event involvement → global observed hosting support	0.147	0.024	0.188	<0.001	[0.100, 0.194]
Winter-sport involvement → global observed hosting support	−0.066	0.027	−0.067	0.014	[−0.118, −0.013]
Global observed hosting support → engagement intentions	**0.380**	**0.024**	**0.416**	**<0.001**	**[0.334, 0.427]**
**Local-instrumental benefits → engagement intentions**	**0.095**	**0.041**	**0.060**	**0.019**	**[0.016, 0.175]**
**Event involvement → engagement intentions**	**0.290**	**0.028**	**0.406**	**<0.001**	**[0.235, 0.345]**
**Winter-sport involvement → engagement intentions**	**0.191**	**0.020**	**0.214**	**<0.001**	**[0.152, 0.231]**

*Note*. *N* = 2021. B = unstandardized coefficient; SE = standard error; β = standardized coefficient; CI = confidence interval. Confidence intervals refer to B. For ordered outcomes, unstandardized coefficients are expressed on the underlying latent-response scale. Bold identifies focal hypothesized paths; statistically significant nonfocal residual paths are not interpreted as substantive findings. The local-instrumental-benefit-to-engagement path was not statistically significant in the age-restricted sensitivity model (β = 0.041, *p* = 0.115). EI = event involvement; LB = local-instrumental benefits; SB = symbolic-social benefits; PC = perceived costs; ENG = engagement intentions.

**Table 5:** WLSMV indirect associations in the overall sample.

Indirect Association	Standardized Estimate	b	95% CI	pBH
SB → Observed support → ENG	0.149	0.301	[0.245, 0.356]	<0.001
PC → Observed support → ENG	−0.050	−0.089	[−0.123, −0.054]	<0.001
EI → SB → Observed support → ENG	0.081	0.058	[0.045, 0.071]	<0.001
EI → PC → Observed support → ENG	0.014	0.010	[0.006, 0.014]	<0.001
EI → LB → ENG	0.037	0.027	[0.004, 0.049]	0.019

*Note*. WLSMV delta-method confidence intervals refer to unstandardized indirect estimates. CI = confidence interval; pBH = Benjamini–Hochberg-adjusted *p* value. The pBH values were adjusted together across the five tests.

**Table 6:** City-specific structural pathways and between-city tests.

Path	Shanghai β	Shenyang β	Δχ^2^(1)	pFDR
Event involvement → local benefits	0.789	0.501	38.26	<0.001
Winter-sport involvement → local benefits †	−0.117	0.017	6.86	0.018
Event involvement → symbolic benefits	0.666	0.481	4.71	0.042
Winter-sport involvement → symbolic benefits †	−0.252	0.019	15.71	<0.001
Event involvement → perceived costs	−0.432	−0.204	15.04	<0.001
Winter-sport involvement → perceived costs †	0.143	0.090	0.69	0.436
Symbolic benefits → global observed hosting support	0.344	0.379	0.59	0.444
Perceived costs → global observed hosting support	−0.190	−0.025	15.34	<0.001
Event involvement → global observed hosting support	0.208	0.178	0.92	0.395
Winter-sport involvement → global observed hosting support †	−0.024	−0.143	17.94	<0.001
Global observed hosting support → engagement	0.329	0.505	22.50	<0.001
Local benefits → engagement	0.096	0.001	3.14	0.097
Event involvement → engagement	0.508	0.335	5.89	0.024
Winter-sport involvement → engagement	0.139	0.279	6.13	0.023

*Note*. WLSMV standardized coefficients are reported. All 14 tested structural comparisons are shown. Between-city differences were tested with nested single-path equality constraints and scaled chi-square difference tests, each with 1 df; *p* values were adjusted across the 14 comparisons using the Benjamini–Hochberg procedure. pFDR = false-discovery-rate-adjusted *p* value. A difference in whether a path is individually significant is not, by itself, evidence of a between-city difference. † Conditional winter-sport paths are reported for completeness but are not interpreted substantively because of sign reversals and suppression-prone residual patterns.

**Table 7:** Summary of hypothesis tests.

Hypothesis/RQ	Theoretical Relation	Conclusion
H1	Event involvement → benefit appraisals (+) and perceived costs (−)	Supported across all three appraisal paths
H2	Symbolic-social benefits (+) and perceived costs (−) → hosting support	Supported for the aggregate compact constructs; component-level cost results were specification-sensitive
H3	Hosting support, local-instrumental benefits, and event- and sport-specific involvement → engagement intentions	Supported for support and both involvement pathways; local-benefit path qualified
H4	Symbolic-social benefits (+) and costs (−) → hosting support → engagement	Supported as FDR-adjusted, model-implied cross-sectional indirect associations; not evidence of temporal mediation
RQ1	City differences in structural pathways	Selective exploratory differences identified

## Data Availability

The de-identified data supporting the findings are available from the corresponding author on reasonable request, subject to institutional and ethical review. Unrestricted public release was not covered by the consent procedure. The executable analysis code is provided as Supplementary File S2.

## Supplementary Materials

The following supporting information can be downloaded at: https://www.mdpi.com/article/10.3390/bs16101727/s1, Supplementary File S1, containing Supplementary Note A, Supplementary Methods S1, and Tables S1–S5 (dataset-overlap disclosure; questionnaire items and documented origins; measurement-invariance results; alternative hosting-support and cost specifications; sample-definition sensitivity; and first-order domain-score sensitivity); Supplementary File S2, executable R analysis code.
