# Supplementary material for: Mega-Event Appraisals, Support, and Engagement Intentions Beyond Host Regions: Pre-Event Evidence from Two Non-Host Cities
Source: Behav Sci (Basel). 2026 Sep 23;16(10):1727. doi: 10.3390/bs16101727 (PMC13701636; doi:10.3390/bs16101727)
Supplement: Supplementary file 1 [file behavsci-16-01727-s001.zip › behavsci-4540988_Supplementary_File_S1.pdf]

Supplementary File S1: Supplementary Methods and Tables

Mega-Event Appraisals, Support, and Engagement Intentions Beyond Host Regions: Pre-Event Evidence from Two Non-Host Cities

This file provides item-level documentation, a concise disclosure of overlap with the earlier publication, compact-model and invariance specifications, and sensitivity analyses that complement the main article. Complete city-path comparisons are reported in main-text Table 6; executable analysis code is provided separately as Supplementary File S2.

Supplementary Note A. Relationship to Jiang et al. (2021)

The present article and Jiang et al. (2021) draw on the same broader Beijing 2022 survey project and substantially overlapping 2020 respondent records. Jiang et al. retained 2,022 cases after excluding 22 questionnaires, whereas the present analysis excludes 23 invariant-response cases and retains 2,021. The 31 benefit items, 15 cost items, and direct hosting-support item are shared. Jiang et al. examined perceived impacts in relation to social cohesion and a moderation model; the present article does not analyze social cohesion and instead examines event and winter-sport involvement, hosting support, and five engagement-intention items using ordinal WLSMV, measurement-invariance tests, model-implied indirect products, and formal city-path comparisons. The primary outcomes and structural estimands are therefore distinct despite the shared survey source.

Table S1. Focal Questionnaire Items and Documented Origin

All items used a five-point strongly-disagree to strongly-agree response scale. English wording is a faithful translation of the Chinese questionnaire. The original development archive supports construct-level, not one-to-one item-level, source attribution. Accordingly, the origin column reports the most specific defensible provenance. Positive-impact items referring to the respondent's city used a city-specific label.

ID	Construct/domain	English item wording	Documented origin/role
EI1	Event involvement	Following the Beijing Olympic Winter Games is important to me.	Adapted at construct level from sport/leisure involvement research; contextualized to Beijing 2022 and refined in instrument development
EI2		Following the Beijing Olympic Winter Games is one of my main leisure activities.	Adapted at construct level from sport/leisure involvement research; contextualized to Beijing 2022 and refined in instrument development
EI3		I closely follow information about the Beijing Olympic Winter Games.	Adapted at construct level from sport/leisure involvement research; contextualized to Beijing 2022 and refined in instrument development
EI4		Spending time learning about the Beijing Olympic Winter Games is worthwhile to me.	Adapted at construct level from sport/leisure involvement research; contextualized to Beijing 2022 and refined in instrument development
EI5		My attention to the Beijing Olympic Winter Games reflects my interests.	Adapted at construct level from sport/leisure involvement research; contextualized to Beijing 2022 and refined in instrument development
EI6		My attention to the Beijing Olympic Winter Games reflects my values.	Adapted at construct level from sport/leisure involvement research; contextualized to Beijing 2022 and refined in instrument development

ID	Construct/domain	English item wording	Documented origin/role
WSI1	Winter-sport involvement	I am a winter-sport enthusiast.	Sport-involvement identity and participation-frequency indicators; contextualized in instrument development
WSI2		I participate in winter sports regularly.	Sport-involvement identity and participation-frequency indicators; contextualized in instrument development
B1	Tourism benefits	Beijing 2022 will help develop tourism in [respondent's city].	Resident-impact literature synthesis plus Shanghai/Shenyang focus-group refinement; pilot item analysis
B2		Beijing 2022 will help upgrade the tourism environment in [respondent's city].	Resident-impact literature synthesis plus Shanghai/Shenyang focus-group refinement; pilot item analysis
B3		The visibility of [respondent's city] will increase because of Beijing 2022.	Resident-impact literature synthesis plus Shanghai/Shenyang focus-group refinement; pilot item analysis
B4		Beijing 2022 will help [respondent's city] develop new tourist attractions.	Resident-impact literature synthesis plus Shanghai/Shenyang focus-group refinement; pilot item analysis
B5	Economic/environmental development	Beijing 2022 will help optimize the industrial structure of [respondent's city].	Resident-impact literature synthesis plus Shanghai/Shenyang focus-group refinement; pilot item analysis
B6		Beijing 2022 will help increase employment opportunities in [respondent's city].	Resident-impact literature synthesis plus Shanghai/Shenyang focus-group refinement; pilot item analysis
B7		Beijing 2022 will help promote economic development in [respondent's city].	Resident-impact literature synthesis plus Shanghai/Shenyang focus-group refinement; pilot item analysis
B8		Beijing 2022 will help increase residents' environmental awareness.	Resident-impact literature synthesis plus Shanghai/Shenyang focus-group refinement; pilot item analysis
B9		Beijing 2022 will help advance green technologies.	Resident-impact literature synthesis plus Shanghai/Shenyang focus-group refinement; pilot item analysis
B10	Social-capital/psychological gains	Beijing 2022 will help improve skills and knowledge for organizing major events.	Resident-impact literature synthesis plus Shanghai/Shenyang focus-group refinement; pilot item analysis

ID	Construct/domain	English item wording	Documented origin/role
B11		Beijing 2022 will help strengthen social cohesion.	Resident-impact literature synthesis plus Shanghai/Shenyang focus-group refinement; pilot item analysis
B12		Beijing 2022 will help promote volunteering.	Resident-impact literature synthesis plus Shanghai/Shenyang focus-group refinement; pilot item analysis
B13		Beijing 2022 will help promote national unity.	Resident-impact literature synthesis plus Shanghai/Shenyang focus-group refinement; pilot item analysis
B14		Beijing 2022 will help increase national pride.	Resident-impact literature synthesis plus Shanghai/Shenyang focus-group refinement; pilot item analysis
B15		Beijing 2022 will help improve civic conduct among residents.	Resident-impact literature synthesis plus Shanghai/Shenyang focus-group refinement; pilot item analysis
B16		Beijing 2022 provides an opportunity to enjoy elite sport competition without leaving China.	Resident-impact literature synthesis plus Shanghai/Shenyang focus-group refinement; pilot item analysis
B17	International cooperation/exchange	Beijing 2022 will help promote international economic and trade cooperation.	Resident-impact literature synthesis plus Shanghai/Shenyang focus-group refinement; pilot item analysis
B18		Beijing 2022 will help promote international cultural exchange.	Resident-impact literature synthesis plus Shanghai/Shenyang focus-group refinement; pilot item analysis
B19		Beijing 2022 will help promote international friendship.	Resident-impact literature synthesis plus Shanghai/Shenyang focus-group refinement; pilot item analysis
B20		Beijing 2022 may create additional diplomatic opportunities.	Resident-impact literature synthesis plus Shanghai/Shenyang focus-group refinement; pilot item analysis
B21	Infrastructure benefits	Beijing 2022 will help improve urban infrastructure in [respondent's city].	Resident-impact literature synthesis plus Shanghai/Shenyang focus-group refinement; pilot item analysis
B22		Beijing 2022 will help improve transport infrastructure.	Resident-impact literature synthesis plus Shanghai/Shenyang focus-group refinement; pilot item analysis

ID	Construct/domain	English item wording	Documented origin/role
B23		Beijing 2022 will help [respondent's city] develop new sport and leisure facilities.	Resident-impact literature synthesis plus Shanghai/Shenyang focus-group refinement; pilot item analysis
B24	National-image benefits	Beijing 2022 will help promote Chinese culture globally.	Resident-impact literature synthesis plus Shanghai/Shenyang focus-group refinement; pilot item analysis
B25		Beijing 2022 will help the world understand China better.	Resident-impact literature synthesis plus Shanghai/Shenyang focus-group refinement; pilot item analysis
B26		Beijing 2022 will help build a positive national image.	Resident-impact literature synthesis plus Shanghai/Shenyang focus-group refinement; pilot item analysis
B27		Beijing 2022 will help enhance China's international standing.	Resident-impact literature synthesis plus Shanghai/Shenyang focus-group refinement; pilot item analysis
B28	Sport-development benefits	Beijing 2022 will help increase public participation in winter sports.	Resident-impact literature synthesis plus Shanghai/Shenyang focus-group refinement; pilot item analysis
B29		Beijing 2022 will help develop competitive winter sport.	Resident-impact literature synthesis plus Shanghai/Shenyang focus-group refinement; pilot item analysis
B30		Beijing 2022 will help develop winter-sport facilities.	Resident-impact literature synthesis plus Shanghai/Shenyang focus-group refinement; pilot item analysis
B31		Beijing 2022 will help disseminate winter-sport knowledge.	Resident-impact literature synthesis plus Shanghai/Shenyang focus-group refinement; pilot item analysis
C1	Living/resource costs	Beijing 2022 will require a very large amount of public funding.	Resident-impact literature synthesis plus Shanghai/Shenyang focus-group refinement; pilot item analysis
C2		Beijing 2022 will increase my everyday living expenses.	Resident-impact literature synthesis plus Shanghai/Shenyang focus-group refinement; pilot item analysis
C3		Beijing 2022 will contribute to inflation.	Resident-impact literature synthesis plus Shanghai/Shenyang focus-group refinement; pilot item analysis

ID	Construct/domain	English item wording	Documented origin/role
C4		After Beijing 2022, many venues will face operational and utilization difficulties.	Resident-impact literature synthesis plus Shanghai/Shenyang focus-group refinement; pilot item analysis
C5		Preparations for Beijing 2022 involve a risk of corruption.	Resident-impact literature synthesis plus Shanghai/Shenyang focus-group refinement; pilot item analysis
C6		Beijing 2022 may make my travel less convenient.	Resident-impact literature synthesis plus Shanghai/Shenyang focus-group refinement; pilot item analysis
C7	Safety risks	Beijing 2022 may increase crime.	Resident-impact literature synthesis plus Shanghai/Shenyang focus-group refinement; pilot item analysis
C8		Beijing 2022 may encourage disruptive fan behavior.	Resident-impact literature synthesis plus Shanghai/Shenyang focus-group refinement; pilot item analysis
C9		Beijing 2022 may create safety hazards.	Resident-impact literature synthesis plus Shanghai/Shenyang focus-group refinement; pilot item analysis
C10	Pollution risks	Beijing 2022 may damage the natural environment.	Resident-impact literature synthesis plus Shanghai/Shenyang focus-group refinement; pilot item analysis
C11		Beijing 2022 may increase air pollution.	Resident-impact literature synthesis plus Shanghai/Shenyang focus-group refinement; pilot item analysis
C12		Beijing 2022 may generate large amounts of waste.	Resident-impact literature synthesis plus Shanghai/Shenyang focus-group refinement; pilot item analysis
C13	Disease/health risks	Hosting Beijing 2022 may increase the spread of infectious diseases.	Resident-impact literature synthesis plus Shanghai/Shenyang focus-group refinement; pilot item analysis
C14		Beijing 2022 may increase opportunities for HIV transmission.	Resident-impact literature synthesis plus Shanghai/Shenyang focus-group refinement; pilot item analysis
C15		Beijing 2022 may increase the risk of food poisoning.	Resident-impact literature synthesis plus Shanghai/Shenyang focus-group refinement; pilot item analysis

ID	Construct/domain	English item wording	Documented origin/role
A1	Alternative evaluative item	The benefits of the Beijing Olympic Winter Games exceed the costs.	Resident-support literature; retained only in support sensitivity specifications
A2R		Hosting the Olympic Winter Games is a waste of money. (Reverse scored)	Resident-support literature; retained only in support sensitivity specifications
SUP	Global observed hosting support	I support hosting the Beijing Olympic Winter Games.	Direct face-valid hosting-support item; primary observed ordered variable
ENG1	Event-related engagement intention	I will watch television coverage of Beijing 2022.	Behavioral-intention item pool adapted to Beijing 2022; refined through pilot analysis
ENG2		I am willing to attend Beijing 2022 competitions in person.	Behavioral-intention item pool adapted to Beijing 2022; refined through pilot analysis
ENG3		I will recommend that relatives or friends watch Beijing 2022.	Behavioral-intention item pool adapted to Beijing 2022; refined through pilot analysis
ENG4		I am willing to volunteer for Beijing 2022.	Behavioral-intention item pool adapted to Beijing 2022; refined through pilot analysis
ENG5		I am willing to purchase Beijing 2022-related souvenirs.	Behavioral-intention item pool adapted to Beijing 2022; refined through pilot analysis

Supplementary Methods S1. Exact Compact-Model and Invariance Specification

Software: R 4.4.1; lavaan 0.6-19. Estimator: WLSMV; parameterization: theta; mean structures included. Ordered variables were EI1–EI6, SUP, and ENG1–ENG5. Continuous variables were WSI and the seven benefit and four cost domain scores. The measurement-only invariance sequence omitted the single observed SUP item because it is not a multi-indicator latent factor; its thresholds were constrained in the strict-equality structural model. With std.lv left at its default FALSE, lavaan used marker-indicator identification. No correlated item residuals were added. Equality constraints were imposed sequentially on loadings; ordered-item thresholds and continuous-indicator intercepts; and residual variances. A decrease in CFI greater than .010 or an increase in RMSEA greater than .015 was treated as evidence against invariance (Chen, 2007). Robust nested comparisons used lavTestLRT. Exact executable syntax is supplied in Supplementary File S2.

Sample and model checks. The platform supplied 2,044 complete questionnaires (Shanghai, 1,020; Shenyang, 1,024). The invariant-response rule excluded 23 cases (Shanghai, 5; Shenyang, 18), leaving 2,021 cases. No retained 83-item response vector was an exact duplicate. Every reported primary, alternative-support, cost, sample-definition, and multi-group model converged and passed lavaan's post-estimation admissibility check. The unrotated first component across 60 focal items accounted for 24.41% of variance, and score-level variance-inflation factors ranged from 1.06 to 1.90; neither diagnostic rules out common-method bias or conditional suppression.

```
measurement_model <- '
  EI =~ EI1 + EI2 + EI3 + EI4 + EI5 + EI6
  LB =~ tourism + econ_env + infrastructure
  SB =~ soc_psych + international + national_image + sport_development
  PC =~ living_resource + safety + pollution + health
  ENG =~ ENG1 + ENG2 + ENG3 + ENG4 + ENG5
'
```

```
fit_configural <- cfa(measurement_model, data=dat, group='city', estimator='WLSMV',
  parameterization='theta', ordered=ordered_vars, meanstructure=TRUE)
fit_metric <- cfa(..., group.equal='loadings')
fit_scalar <- cfa(..., group.equal=c('loadings','thresholds','intercepts'))
fit_strict <- cfa(..., group.equal=c('loadings','thresholds','intercepts','residuals'))
lavTestLRT(previous_model, more_restricted_model) # Satorra (2000) scaled difference test
```

Table S2. Full Measurement-Invariance Results

Level	Scaled χ^2	df	CFI	TLI	RMSEA	SRMR	ΔCFI	ΔRMSEA	Robust $\Delta\chi^2$	Δ df	p	Converged/admissible
Configural	1924.54	398	0.931	0.920	0.062	0.040	—	—	—	—	—	Yes/Yes
Metric	1781.50	415	0.939	0.932	0.057	0.043	+0.007	-0.005	72.50	17	<.001	Yes/Yes
Scalar	1975.06	454	0.932	0.930	0.058	0.042	-0.007	+0.000	123.30	39	<.001	Yes/Yes
Strict	1912.34	476	0.935	0.937	0.055	0.043	+0.004	-0.003	48.49	22	<.001	Yes/Yes

Note. Robust nested comparisons used lavaan's Satorra (2000) scaled difference test. All chi-square differences were statistically significant; the invariance decision followed the incremental-fit criteria specified in Methods (no CFI decrease > .010 and no RMSEA increase > .015; Chen, 2007). Δ indices were calculated from unrounded fit values.

Table S3. Robustness to Alternative Hosting-Support and Cost Specifications

Specification	Relevant model fit	Focal coefficient estimates
Single observed support	$\chi^2(236) = 2030.92$; CFI = 0.925; TLI = 0.912; RMSEA = 0.061; SRMR = 0.038	SB → SUP: B = 0.790, SE = 0.056, β = 0.359, p < .001, 95% CI [0.680, 0.901]<br>PC → SUP: B = -0.233, SE = 0.046, β = -0.120, p < .001, 95% CI [-0.323, -0.142]<br>EI → SUP: B = 0.147, SE = 0.024, β = 0.188, p < .001, 95% CI [0.100, 0.194]<br>WSI → SUP: B = -0.066, SE = 0.027, β = -0.067, p = .014, 95% CI [-0.118, -0.013]<br>SUP → ENG: B = 0.380, SE = 0.024, β = 0.416, p < .001, 95% CI [0.334, 0.427]
Three-item composite	$\chi^2(236) = 2033.15$; CFI = 0.924; TLI = 0.911; RMSEA = 0.061; SRMR = 0.039	SB → SUP3: B = 0.511, SE = 0.030, β = 0.381, p < .001, 95% CI [0.451, 0.570]<br>PC → SUP3: B = -0.286, SE = 0.025, β = -0.246, p < .001, 95% CI [-0.335, -0.237]<br>EI → SUP3: B = 0.045, SE = 0.013, β = 0.095, p < .001, 95% CI [0.020, 0.070]<br>WSI → SUP3: B = -0.041, SE = 0.014, β = -0.069, p = .003, 95% CI [-0.069, -0.014]<br>SUP3 → ENG: B = 0.444, SE = 0.032, β = 0.304, p < .001, 95% CI [0.380, 0.507]

Specification	Relevant model fit	Focal coefficient estimates
Three-item latent	$\chi^2(282) = 4202.32$; CFI = 0.845; TLI = 0.821; RMSEA = 0.083; SRMR = 0.058	SB → SUPPORT: B = 0.430, SE = 0.037, β = 0.409, p < .001, 95% CI [0.356, 0.503]<br>PC → SUPPORT: B = -0.210, SE = 0.025, β = -0.232, p < .001, 95% CI [-0.258, -0.162]<br>EI → SUPPORT: B = 0.076, SE = 0.013, β = 0.204, p < .001, 95% CI [0.051, 0.101]<br>WSI → SUPPORT: B = -0.034, SE = 0.013, β = -0.071, p = .013, 95% CI [-0.060, -0.007]<br>SUPPORT → ENG: B = 0.828, SE = 0.079, β = 0.425, p < .001, 95% CI [0.674, 0.982]
Aggregate four domains	—	PC → SUP: B = -0.233, SE = 0.046, β = -0.120, p < .001, 95% CI [-0.323, -0.142]
Four domains separate	—	living_resource → SUP: B = -0.060, SE = 0.040, β = -0.040, p = .131, 95% CI [-0.138, 0.018]<br>safety → SUP: B = -0.020, SE = 0.039, β = -0.016, p = .610, 95% CI [-0.097, 0.057]<br>pollution → SUP: B = 0.047, SE = 0.038, β = 0.039, p = .217, 95% CI [-0.027, 0.120]<br>health → SUP: B = -0.137, SE = 0.036, β = -0.113, p < .001, 95% CI [-0.207, -0.067]
Aggregate without health	—	PC → SUP: B = -0.210, SE = 0.046, β = -0.109, p < .001, 95% CI [-0.301, -0.120]

Note. Fit indices are shown for the three support operationalizations because they estimate parallel overall models. Cost rows are coefficient-focused sensitivity specifications. The three-item latent support model produced similar focal directions but poor overall fit and is not treated as a preferred measurement model.

Table S4. Sample-Definition Sensitivity

Sample/model	Path	β	p	95% CI for B
primary n2021	EI → LB	0.624	<.001	[0.248, 0.314]
	EI → SB	0.546	<.001	[0.171, 0.216]
	EI → PC	-0.284	<.001	[-0.139, -0.089]
	SB → SUP	0.359	<.001	[0.680, 0.901]
	PC → SUP	-0.120	<.001	[-0.323, -0.142]
	SUP → ENG	0.416	<.001	[0.334, 0.427]
	LB → ENG	0.060	.019	[0.016, 0.175]
	EI → ENG	0.406	<.001	[0.235, 0.345]
	WSI → ENG	0.214	<.001	[0.152, 0.231]
all completed n2044	EI → LB	0.637	<.001	[0.254, 0.319]
	EI → SB	0.565	<.001	[0.181, 0.228]
	EI → PC	-0.255	<.001	[-0.126, -0.078]
	SB → SUP	0.382	<.001	[0.714, 0.928]
	PC → SUP	-0.111	<.001	[-0.303, -0.130]
	SUP → ENG	0.419	<.001	[0.343, 0.436]

Sample/model	Path	β	p	95% CI for B
	LB → ENG	0.063	.012	[0.022, 0.180]
	EI → ENG	0.401	<.001	[0.236, 0.346]
	WSI → ENG	0.217	<.001	[0.159, 0.241]
adult n1935	EI → LB	0.632	<.001	[0.251, 0.319]
	EI → SB	0.561	<.001	[0.175, 0.222]
	EI → PC	-0.299	<.001	[-0.148, -0.095]
	SB → SUP	0.369	<.001	[0.706, 0.935]
	PC → SUP	-0.131	<.001	[-0.345, -0.161]
	SUP → ENG	0.421	<.001	[0.330, 0.422]
	LB → ENG	0.041	.115	[-0.015, 0.142]
	EI → ENG	0.428	<.001	[0.245, 0.356]
	WSI → ENG	0.208	<.001	[0.142, 0.221]

Note. The primary model excludes 23 invariant-response cases. The all-completed model retains all 2,044 questionnaires, and the adult model excludes respondents younger than 18 years. The local-instrumental-benefit-to-engagement estimate is the only focal path that becomes nonsignificant in the adult sensitivity sample.

Table S5. First-Order Domain-Score Sensitivity

Model	Outcome	Predictor	β	SE	p	95% CI	R^2
appraisal tourism	tourism	EI	0.459	0.023	<.001	[0.414, 0.505]	0.225
appraisal tourism	tourism	WSI	0.027	0.023	.243	[-0.018, 0.073]	0.225
appraisal econ env	econ_env	EI	0.503	0.022	<.001	[0.460, 0.547]	0.287
appraisal econ env	econ_env	WSI	0.055	0.022	.013	[0.011, 0.099]	0.287
appraisal infrastructure	infrastructure	EI	0.448	0.023	<.001	[0.402, 0.494]	0.211
appraisal infrastructure	infrastructure	WSI	0.020	0.023	.387	[-0.026, 0.066]	0.211
appraisal soc psych	soc_psych	EI	0.461	0.024	<.001	[0.414, 0.507]	0.185
appraisal soc psych	soc_psych	WSI	-0.062	0.024	.009	[-0.109, -0.016]	0.185
appraisal international	international	EI	0.391	0.025	<.001	[0.342, 0.439]	0.127
appraisal international	international	WSI	-0.073	0.025	.003	[-0.121, -0.025]	0.127
appraisal national image	national_image	EI	0.401	0.025	<.001	[0.353, 0.450]	0.139

Model	Outcome	Predictor	β	SE	p	95% CI	R^2
appraisal national image	national_image	WSI	-0.059	0.025	.016	[-0.107, -0.011]	0.139
appraisal sport development	sport_development	EI	0.387	0.024	<.001	[0.340, 0.435]	0.159
appraisal sport development	sport_development	WSI	0.020	0.024	.412	[-0.028, 0.067]	0.159
appraisal living resource	living_resource	EI	-0.180	0.026	<.001	[-0.232, -0.129]	0.026
appraisal living resource	living_resource	WSI	0.042	0.026	.111	[-0.010, 0.093]	0.026
appraisal safety	safety	EI	-0.157	0.026	<.001	[-0.209, -0.106]	0.018
appraisal safety	safety	WSI	0.071	0.026	.007	[0.020, 0.122]	0.018
appraisal pollution	pollution	EI	-0.244	0.026	<.001	[-0.295, -0.194]	0.049
appraisal pollution	pollution	WSI	0.048	0.026	.060	[-0.002, 0.099]	0.049
appraisal health	health	EI	-0.214	0.026	<.001	[-0.265, -0.163]	0.042
appraisal health	health	WSI	0.020	0.026	.430	[-0.030, 0.071]	0.042
support first order domains	SUP	soc_psych	0.172	0.036	<.001	[0.101, 0.243]	0.210
support first order domains	SUP	international	0.039	0.032	.227	[-0.024, 0.102]	0.210
support first order domains	SUP	national_image	0.200	0.033	<.001	[0.135, 0.266]	0.210
support first order domains	SUP	sport_development	-0.044	0.030	.138	[-0.103, 0.014]	0.210
support first order domains	SUP	living_resource	-0.048	0.029	.107	[-0.105, 0.010]	0.210
support first order domains	SUP	safety	-0.029	0.032	.351	[-0.091, 0.032]	0.210
support first order domains	SUP	pollution	0.024	0.032	.447	[-0.039, 0.088]	0.210
support first order domains	SUP	health	-0.039	0.030	.198	[-0.098, 0.020]	0.210
support first order domains	SUP	EI	0.170	0.026	<.001	[0.118, 0.221]	0.210
support first order domains	SUP	WSI	-0.043	0.024	.069	[-0.090, 0.003]	0.210
engagement first order domains	ENG	SUP	0.314	0.017	<.001	[0.282, 0.347]	0.509

Model	Outcome	Predictor	β	SE	p	95% CI	R^2
engagement first order domains	ENG	tourism	-0.042	0.024	.083	[-0.090, 0.006]	0.509
engagement first order domains	ENG	econ_env	0.093	0.026	<.001	[0.042, 0.144]	0.509
engagement first order domains	ENG	infrastructure	0.059	0.023	.010	[0.014, 0.103]	0.509
engagement first order domains	ENG	EI	0.339	0.022	<.001	[0.297, 0.381]	0.509
engagement first order domains	ENG	WSI	0.226	0.019	<.001	[0.190, 0.263]	0.509

Note. Standardized score-level linear models provide a transparent alternative to the compact higher-order WLSMV specification. They are sensitivity analyses, not replacements for the primary ordinal model.
